# A case of overcoming *De Novo* thrombotic microangiopathy associated with hyperacute rejection in a living donor ABO-incompatible kidney transplantation despite severe intraoperative graft injury

**DOI:** 10.1007/s13730-026-01161-y

**Published:** 2026-07-20

**Authors:** Hiroshi Ide, Yusuke Tomita, Masahiro Koizumi, Go Ogura, Michio Nakamura

**Affiliations:** 1 Department of Transplant Surgery, Tokai University School of Medicine, 143 Shimokasuya, Isehara, Kanagawa 259-1193 Japan; 2 Department of Nephrology, Endocrinology, and Metabolism, Tokai University School of Medicine, Isehara, Kanagawa Japan; 3 Department of Pathology, Tokai University School of Medicine, Isehara, Kanagawa Japan

**Keywords:** End-stage renal disease, Kidney transplant, ABO-incompatible, De novo thrombotic microangiopathy, Hyperacute rejection, HLA-DP

## Abstract

A 19-year-old male with end-stage renal disease caused by vesicoureteral reflux, hypertension, and type 2 diabetes mellitus underwent preemptive kidney transplantation using his mother’s left kidney. This was an ABO-incompatible kidney transplant between a recipient with blood type O and a donor with blood type B. Therefore, plasma exchange and rituximab administration were performed preoperatively as desensitization therapy. Immediately after vascular anastomosis, blood flow in the transplanted renal cortex was assessed using ultrasonography, which was deemed adequate. Nonetheless, the urine output did not increase. Ultrasound re-evaluation revealed an elevated vascular resistance index in the transplanted renal cortex, raising hyperacute rejection suspicion. Rejection therapy was initiated before vesicoureteral anastomosis. Because concomitant *de novo* thrombotic microangiopathy (dnTMA) was also suspected, anticoagulant therapy was initiated. By the end of the surgery, the velocity of the renal cortical blood flow had decreased, prompting immediate plasma exchange. Hemodialysis was performed on postoperative day (POD) 0 but was not required thereafter. Serum creatinine levels remained at around 5–6 mg/dL for some time; nevertheless, they began to decrease on POD15, finally improving to 1.36 mg/dL on POD 65. A renal biopsy performed 1 h after reperfusion revealed fibrin thrombi and inflammatory cell infiltrates in the glomerular capillaries (g1), resulting in the diagnosis of dnTMA due to hyperacute rejection. The cause of hyperacute rejection was possibly HLA-DP donor-specific antibody. This report describes a case in which the prompt treatment for dnTMA due to hyperacute rejection preserved graft function.

**Supplementary information:**

The online version contains supplementary material available at 10.1007/s13730-026-01161-y.

## Introduction

*De novo* thrombotic microangiopathy (dnTMA) following kidney transplantation (KTx) is a rare but serious complication that can lead to early graft loss [1–6]. Although ABO-incompatible kidney transplantation (ABOi-KTx) has become increasingly feasible with modern desensitization protocols, dnTMA with hyperacute rejection during surgery has rarely been reported, and its optimal management remains unclear. Herein, we report a case of successful living-donor ABOi-KTx despite severe intraoperative graft injury due to dnTMA with hyperacute rejection.

## Case report

**Fig. 1:**
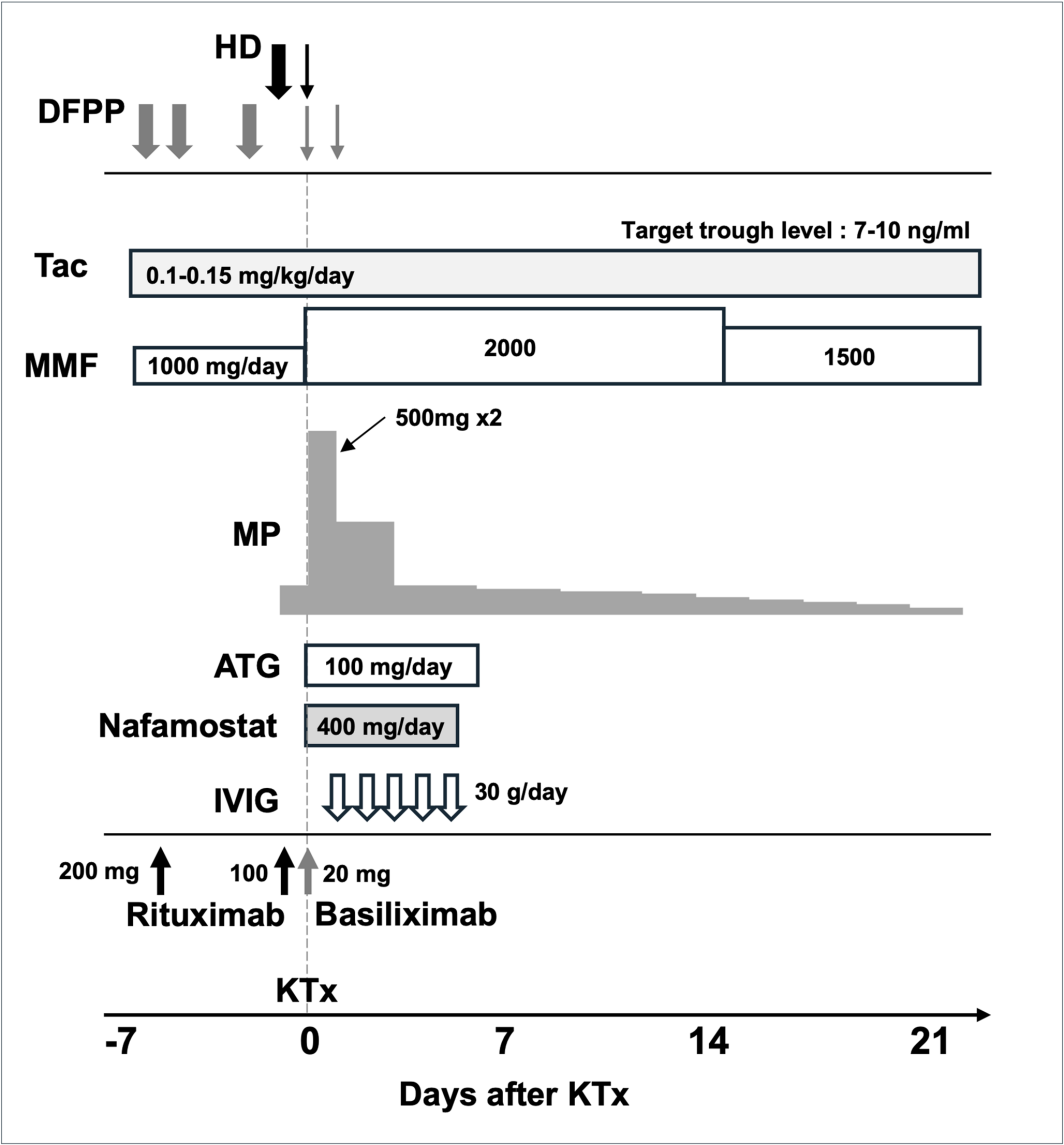
The perioperative treatment included comprehensive rejection management, especially after surgery. In addition to a standard protocol of three preoperative double filtration plasmapheresis (DFPP) sessions, anti-human thymocyte immunoglobulin, nafamostat mesylate, and intravenous immunoglobulin were administered consecutively for 7, 6, 5 days, respectively, after KTx. Two additional DFPP treatments were performed postoperatively on POD 0 and POD 1. DFPP: double filtration plasmapheresis (Thick gray arrows indicate DFPP before KTx. Thin gray arrows indicate DFPP after KTx.), HD: hemodialysis (Thick black arrows indicate HD before KTx. Thin black arrows indicate HD after KTx.), Tac: tacrolimus, MMF: mycophenolate mofetil, MP: methylprednisolone, ATG: anti-human thymocyte immunoglobulin, Nafamostat: nafamostat mesylate, IVIG: intravenous immunoglobulin, KTx: kidney transplantation

**Fig. 2:**
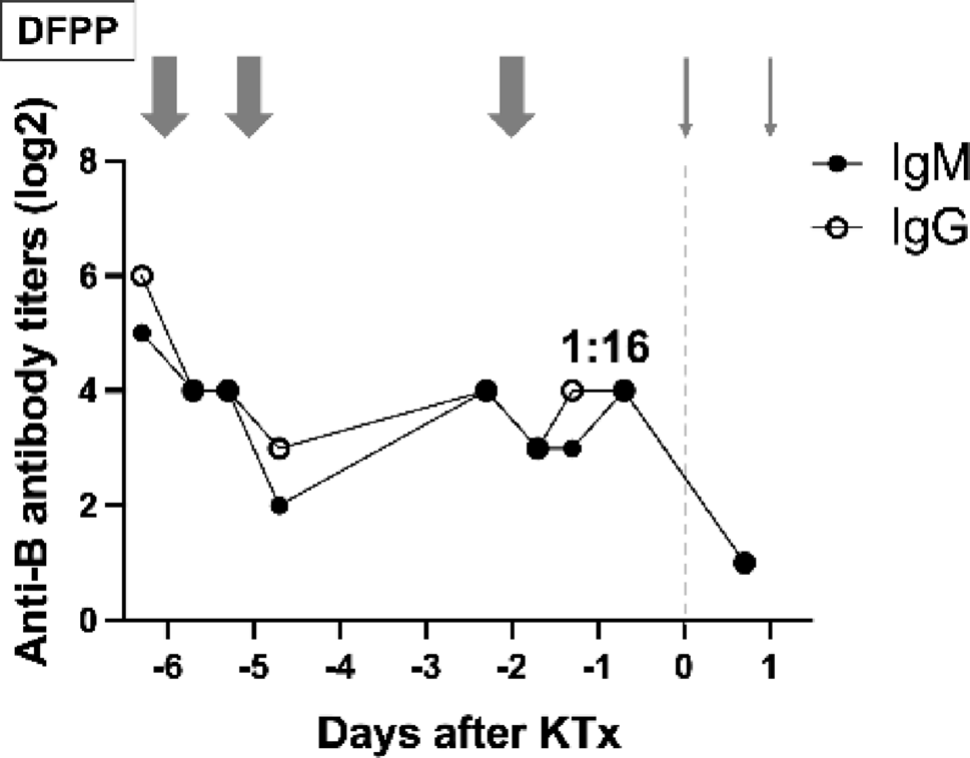
Anti-B antibody titers were measured around the time of the kidney transplantation. Prior to surgery, three sessions of double filtration plasmapheresis (DFPP) were performed as plasma exchange therapy, resulting in 1:16 in immunoglobulin M and immunoglobulin G antibody titers. Postoperatively, suspected hyperacute rejection led to two additional DFPP sessions on postoperative day (POD) 0 and POD 1. DFPP: double filtration plasmapheresis (Thick gray arrows indicate DFPP before KTx. Thin gray arrows indicate DFPP after KTx.), IgM: immunoglobulin M, IgG: immunoglobulin G, KTx: kidney transplantation

The recipient underwent surgery for vesicoureteral reflux (VUR) in the right kidney at 2 years of age, although the details are unknown. Since then, he visited the hospital for VUR, hypertension (HTN), type 2 diabetes mellitus (T2DM), hyperuricemia, and dyslipidemia. His renal function gradually worsened, and at 19 years he underwent preemptive kidney transplantation for end-stage renal disease using his mother’s left kidney; VUR, HTN, and T2DM were the recipient’s primary diseases. The donor was 57 years old, and the estimated glomerular filtration rate (eGFR) by cystatin C before transplantation was 90.1 mL/min. At transplantation, the patient had obesity and weighed 100 kg with a body mass index of 31.2. The recipient and donor had blood types O and B, respectively, demonstrating incompatibility (Table 1). Tacrolimus, a calcineurin inhibitor, and mycophenolate mofetil were orally administered before surgery (Fig. 1). Furthermore, according to our desensitization protocol for ABOi-KTx, three sessions of double filtration plasmapheresis (DFPP) were performed for plasma exchange, and 300 mg rituximab was administered preoperatively (Fig. 1). The anti-B antibody titers became 1:16 on the day before surgery and did not rebound significantly (Fig. 2).

**Table 1:**
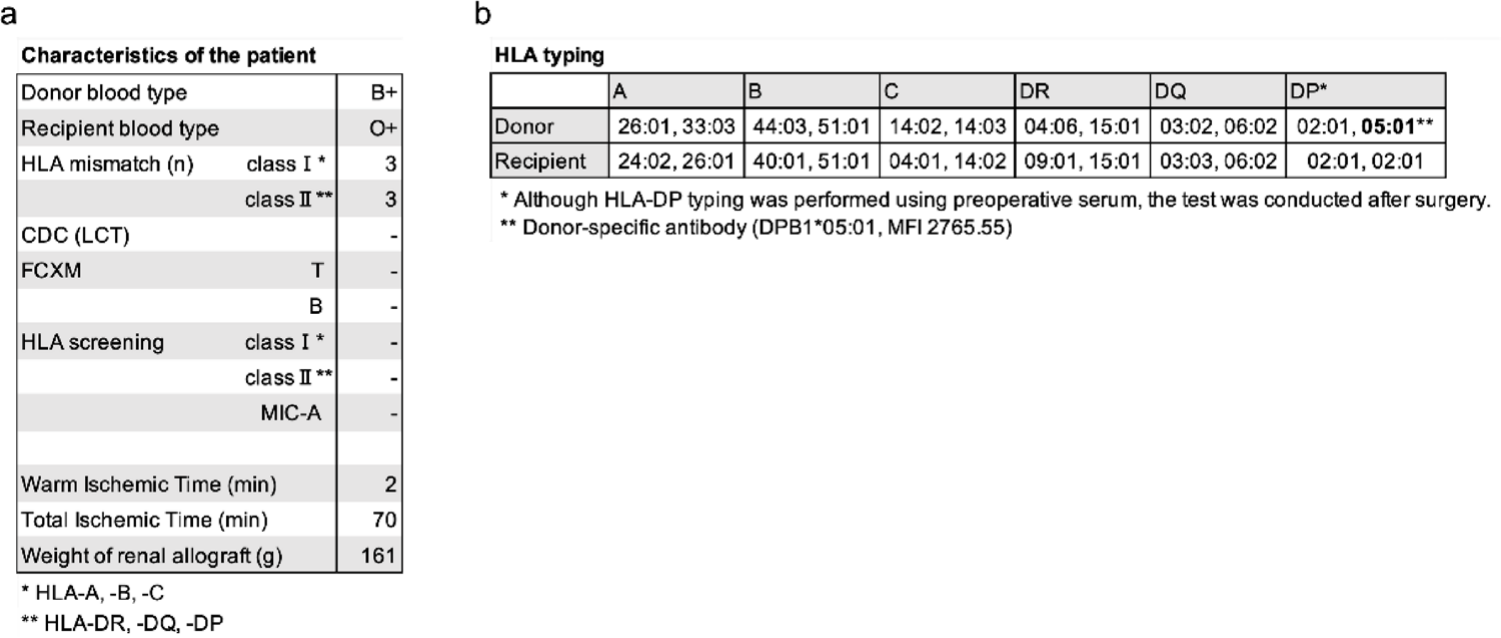
The patient characteristics indicated blood type incompatibility as an immunological risk. (a) Characteristics of the patient. (b) HLA typing

**Fig. 3:**
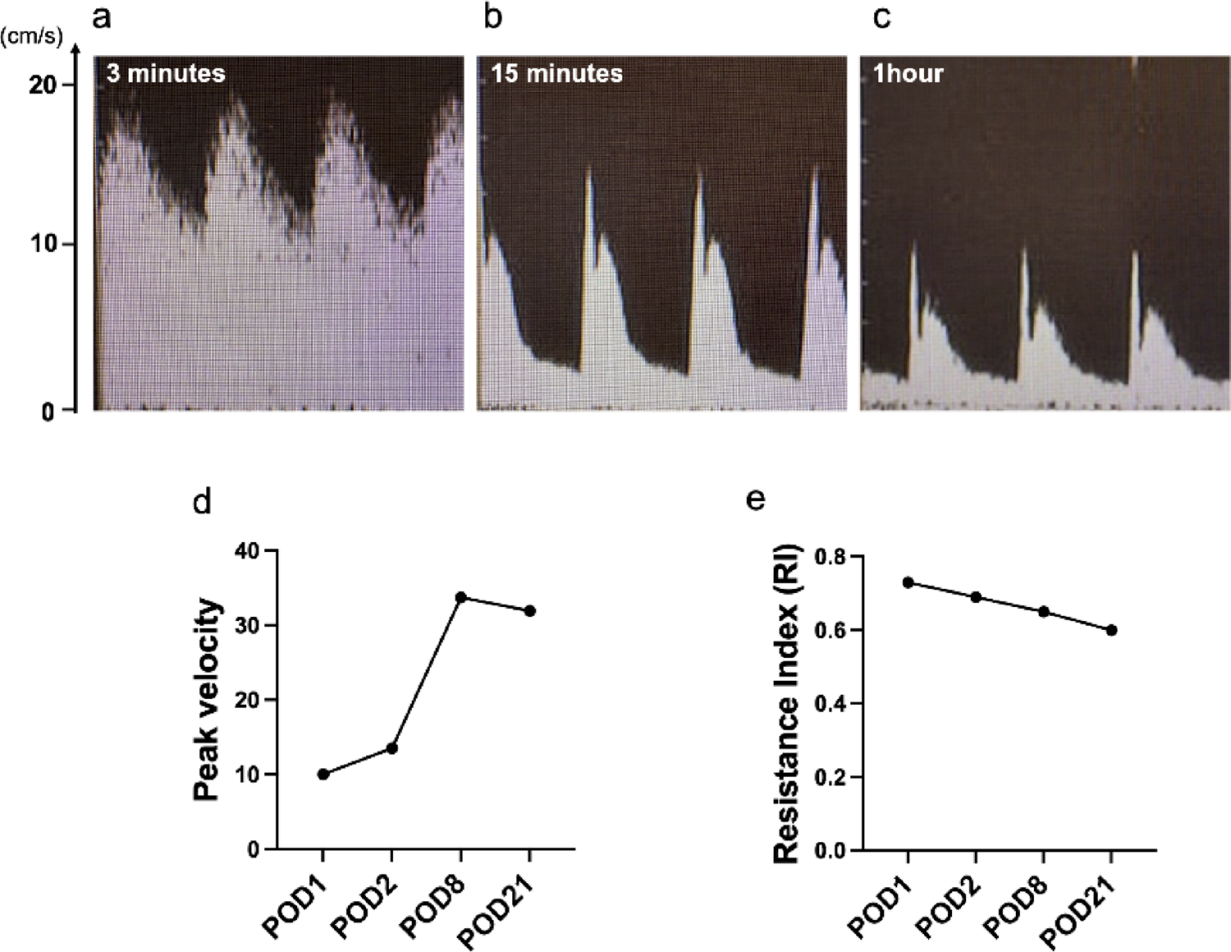
The ultrasound waveform of interlobular artery blood flow in the transplanted renal cortex gradually deteriorated following vascular anastomosis during surgery (**a–c**): **a** 3 min after blood flow resumed; **b** 15 min after blood flow resumed; **c** 1 h after blood flow resumed. Postoperatively, peak systolic velocity and resistance index gradually improved (**d** and** e**, respectively)

Twenty milligrams of basiliximab (anti-CD25 monoclonal antibody) were administered at the start of surgery, followed by 500 mg of methylprednisolone during vascular anastomosis. After resuming blood flow following vascular anastomosis between the recipient’s right external iliac vessels and donor renal vessels, ultrasonography revealed that the systolic and diastolic velocities in the arterial blood flow of the transplanted renal cortex were stable and blood flow to the transplanted kidney was adequate (Fig. 3a). However, because the urine output did not increase, another ultrasound was performed before vesicoureteral anastomosis, which especially showed decreased diastolic blood velocity, and hyperacute rejection of the transplanted kidney was clinically suspected (Fig. 3b). Intraoperatively, an additional 500 mg of methylprednisolone and heparin were administered; nonetheless, there was no improvement in the transplant renal blood flow. Therefore, anti-human thymocyte immunoglobulin was administered (Fig. 1). Following vesicoureteral anastomosis, ultrasound revealed decreased systolic and diastolic blood velocities in the arterial blood flow of the transplanted renal cortex (Fig. 3c), as well as deterioration in the color tone and tension of the transplanted kidney. Therefore, dnTMA with hyperacute rejection was considered, and intravenous immunoglobulin (IVIG) and nafamostat mesylate were administered on the day of surgery, along with additional DFPP (Fig. 1).

**Fig. 4:**
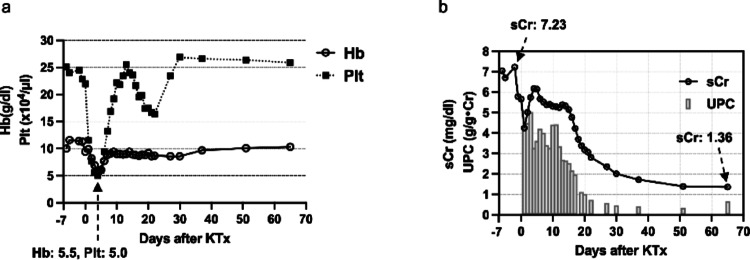
**a** Laboratory data for hemoglobin and platelets showed the greatest decline on postoperative day (POD) 4, then improved. Hb: hemoglobin, Plt: platelet. **b** Laboratory data for sCr, and UPC gradually improved from POD15. sCr: serum creatinine, UPC: urine protein creatinine ratio, KTx: kidney transplantation

On postoperative day (POD) 4, the patient experienced a decrease in platelets (5.0 × 10^4^/µl) and abdominal wall bleeding, resulting in decreased hemoglobin (5.5 g/dl) (Fig. 4a). Nonetheless, there was no bleeding around the transplanted kidney or the vesicoureteral anastomosis that required reoperation.

Anti-human thymocyte immunoglobulin, nafamostat mesylate, and IVIG were administered consecutively for 7, 6, 5 days, respectively. Serum creatinine (sCr) remained at around 5–6 mg/dL without hemodialysis for approximately 2 weeks after transplantation, except on POD 0 (Fig. 4b). Nevertheless, the systolic (peak) and diastolic velocities and resistance indices in the arterial blood flow of the transplanted renal cortex improved on ultrasonography on POD 8 (Fig. 3d, e). sCr levels began to decrease on POD15 (Fig. 4b). The patient was discharged on POD 22 with sCr and blood urea nitrogen (BUN) levels of 2.81 mg/dL and 52 mg/dL, respectively (Fig. 4b). Additionally, because the donor and recipient were cytomegalovirus-immunoglobulin g-positive and -negative, respectively, Retermovir administration to the patient was initiated prior to discharge; subsequently, he was monitored in an outpatient clinic. On POD 65, we confirmed that sCr and BUN levels had decreased to 1.36 and 13 mg/dL, respectively (Fig. 4b).

**Fig. 5:**
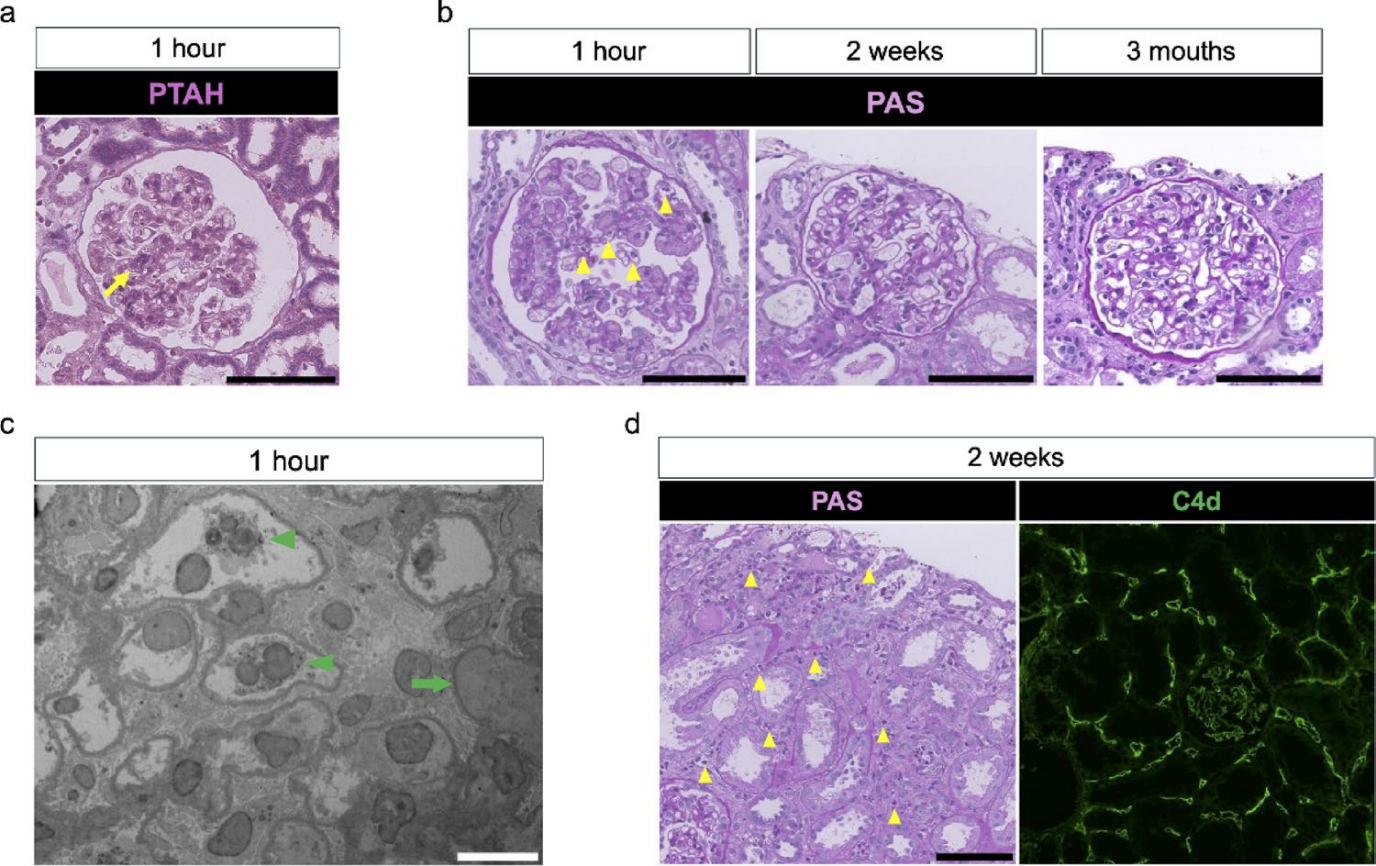
The pathological images from biopsies of the transplanted renal cortex. **a** Phosphotungstic acid–hematoxylin (PTAH) staining at 1 h after vascular anastomosis during surgery demonstrates a prominent fibrin thrombus within the glomerular capillary (yellow arrow). Scale bars: 100 μm. **b** Periodic acid–Schiff (PAS) staining at three time points (1 h after vascular anastomosis, 2 weeks, and 3 months after the kidney transplantation). The panel at 1 h shows fibrin thrombus and inflammatory cell infiltrates in the glomerular capillaries (yellow arrowheads indicate representative inflammatory cell infiltrates), whereas the remaining panels do not. Scale bar: 100 μm. **c** In the 1-hour biopsy, electron micrographs showed thrombi in some glomerular capillaries (green arrow). Furthermore, many capillaries displayed severe endothelial damage, with cellular detachment from the basement membrane and concurrent neutrophil infiltration (green arrowhead). Scale bar: 10 μm. **d** Left: PAS staining exhibits inflammatory cell infiltrates in the peritubular capillaries (yellow arrowheads indicate the representative cells). Scale bar: 100 μm. Right: complement component 4d (C4d) immunostaining is positive in the peritubular capillaries

The pathological findings from biopsies of the transplanted renal cortex were as follows. The biopsy specimen obtained 1 h after vascular anastomosis during surgery revealed fibrin thrombi and glomerulitis (g1) in the glomerular capillaries (Fig. 5a, b), leading to a dnTMA diagnosis triggered by hyperacute rejection; nonetheless, it was C4d-negative in the peritubular capillaries (data not shown). Despite preserved staining intensity for the endothelial markers CD31 and CD34 (data not shown), detailed electron microscopy revealed substantial endothelial injury associated with neutrophil infiltration within the glomerular capillaries (Fig. 5c). Conversely, the fibrin thrombi disappeared from the biopsy specimen 2 weeks following KTx (Fig. 5b). Furthermore, tissue sections obtained 2 weeks post-KTx revealed peritubular capillaritis (ptc1) and C4d positivity in the peritubular capillaries (Fig. 5d). Therefore, although the fibrin thrombi and glomerulitis had improved by 2 weeks post-KTx, peritubular capillaritis may have been the main pathological finding at that time. Finally, both glomerulitis and peritubular capillaritis improved 3 months after KTx (Fig. 5b).

## Discussion

DnTMA with hyperacute rejection is a serious complication that significantly affects graft survival. Although improvement from the complication takes approximately 2 weeks, this report is valuable for demonstrating the favorable outcomes of early diagnosis and treatment.

In this case, vascular anastomosis for KTx was performed without surgical complications. Initial urine output was noted shortly after vascular anastomosis; nevertheless, this was followed by a decrease to oliguric levels. Blood vessels at the anastomotic site in the right iliac fossa were not compressed physically by the surrounding structures. The absence of obvious renal artery stenosis due to displaced positioning of the graft was confirmed through visual inspection and palpation during surgery. That was further confirmed by postoperative ultrasound examination (Supplemental Fig. S1). The systolic pressure was maintained at around 150mmHg after vascular anastomosis. Sufficient intravenous fluids were administered during surgery. Blood flow to the transplanted kidney was adequate immediately after declamping following vascular anastomosis (Fig. 3a). Therefore, the decreased blood flow in the transplanted renal cortex is unlikely due to anatomical causes and led to suspicion of hyperacute rejection, prompting initiation of treatment for rejection during surgery. As the graft did not respond to additional steroid administration, concomitant dnTMA was also considered. To differentiate dnTMA from atypical hemolytic uremic syndrome (aHUS), we examined haptoglobin levels, which remained normal and did not indicate hemolysis; therefore, the diagnosis of aHUS was unlikely. Furthermore, genetic testing performed after surgery failed to support a diagnosis of aHUS.

The preoperative anti-B antibody titers decreased to 1:16, with no significant rebound even after plasma exchange. The decrease could be related to the lack of C4d staining at the 1-hour biopsy. Additionally, the short time between the declamping and the biopsy may explain it. The Banff classification allows for the diagnosis of antibody-mediated rejection (ABMR) in the absence of ptc C4d staining [7]. In fact, cases of C4d-negative ABMR have been reported [8]. Following vascular anastomosis and declamping, an antigen–antibody reaction occurred intraoperatively in the endothelial cells of the glomerular capillaries, resulting in damage of endothelial cells and microthrombosis in the glomerular capillaries. The pathological outcomes supported this finding (Fig. 5a, b, c). Therefore, we concluded that the decreased blood flow in the transplanted renal cortex resulted from ABMR during surgery.

Additionally, the recipient exhibited blood type O on ABOi-KTx. Recipients with blood type O have been reported to have high anti-blood-type antibody titers, associated with early graft loss and antibody-mediated rejection [9]. Furthermore, anti-A and anti-B antibodies with complement-binding activity may cause hyperacute rejection, even at low antibody titers [10]. Further research and additional cases are needed to conclude whether type O recipients in ABOi-KTx are associated with ABMR.

The HLA antibody screening (LABScreen Mixed) was negative, the preformed donor-specific antibody (DSA) test was also negative preoperatively. Nonetheless, HLA-DP typing is generally not performed in kidney transplant recipients and donors, although we performed HLA-A, -B, -C, -DR, and -DQ typing. To identify the rejection cause, we conducted HLA-DP typing on the recipient and donor and anti-HLA antibody testing (LABScreen Single Antigen) postoperatively using the preoperative recipient’s serum collected before plasma exchange, revealing that the anti-HLA-DP antibody was DSA (DPB1*05:01, mean fluorescence intensity: 2765.55). Notably, HLA-DP DSAs are associated with ABMR [11–13]. The clinical significance of anti-HLA-DP DSAs has been increasingly highlighted [11–15]. If the presence of anti-HLA-DP DSA had been known preoperatively, IVIG could have been administered as preoperative desensitization therapy, which might have prevented hyperacute rejection. However, since the MFI value is not high, we cannot definitively conclude that the anti-HLA-DP DSA is a cause of ABMR.

Postoperatively, hemodialysis was performed on POD 0, and the sCr did not worsen from preoperative levels. Subsequently, ultrasonography consistently demonstrated no renal artery stenosis or obvious fluid retention around the bladder or transplanted kidney. Blood flow to the transplanted kidney improved by POD 8, and sCr, BUN, proteinuria, and other parameters gradually improved from POD 15. Interestingly, pathological findings showed that the fibrin thrombi detected in the 1-h biopsy had disappeared by the 2-week biopsy, and glomerulitis had improved. Early rejection treatment and anticoagulation therapy may have maintained the injury within a reversible range, contributing to improvement in graft function.

Furthermore, because it was a preemptive transplant with spontaneous urine output maintained postoperatively, evaluating graft function based solely on urine output is challenging. Abdominal wall hemorrhage was caused by DFPP, anticoagulant therapy, and decreased platelet count, which led to anemia. The low platelet count may have been caused by side effects of anti-human thymocyte immunoglobulin or consumption due to glomerular thrombosis. To detect the above issues at an early stage, routine daily transplant-kidney ultrasound and blood tests proved effective.

While dnTMA with hyperacute rejection is often associated with a poor prognosis, particularly when it occurs during surgery, this case demonstrated that prompt recognition and intervention can preserve graft function, even in high-risk settings such as ABOi-KTx. Further studies are warranted to clarify optimal management strategies for this rare but severe complication.

## Supplementary information

Below is the link to the electronic supplementary material.

Supplementary Material 1
