# Supplementary material for: A case of overcoming De Novo thrombotic microangiopathy associated with hyperacute rejection in a living donor ABO-incompatible kidney transplantation despite severe intraoperative graft injury
Source: CEN Case Rep. 2026 Jul 20;15(4):120. doi: 10.1007/s13730-026-01161-y (PMC13385312; doi:10.1007/s13730-026-01161-y)
Supplement: Supplementary file 1 — Supplementary Material 1 [file 13730_2026_1161_MOESM1_ESM.pdf]

CEN Case Reports

Supplementary material

A Case of Overcoming *De Novo* Thrombotic Microangiopathy Associated with Hyperacute Rejection in a Living Donor ABO-Incompatible Kidney Transplantation Despite Severe Intraoperative Graft Injury

Authors:

Hiroshi Ide*, Yusuke Tomita, Masahiro Koizumi, Go Ogura, Michio Nakamura

Affiliation and e-mail address of the corresponding author*:

Department of Transplant Surgery, Tokai University School of Medicine, 143 Shimokasuya, Isehara, Kanagawa 259-1193, Japan.

E-mail: h-ide@tokai.ac.jp

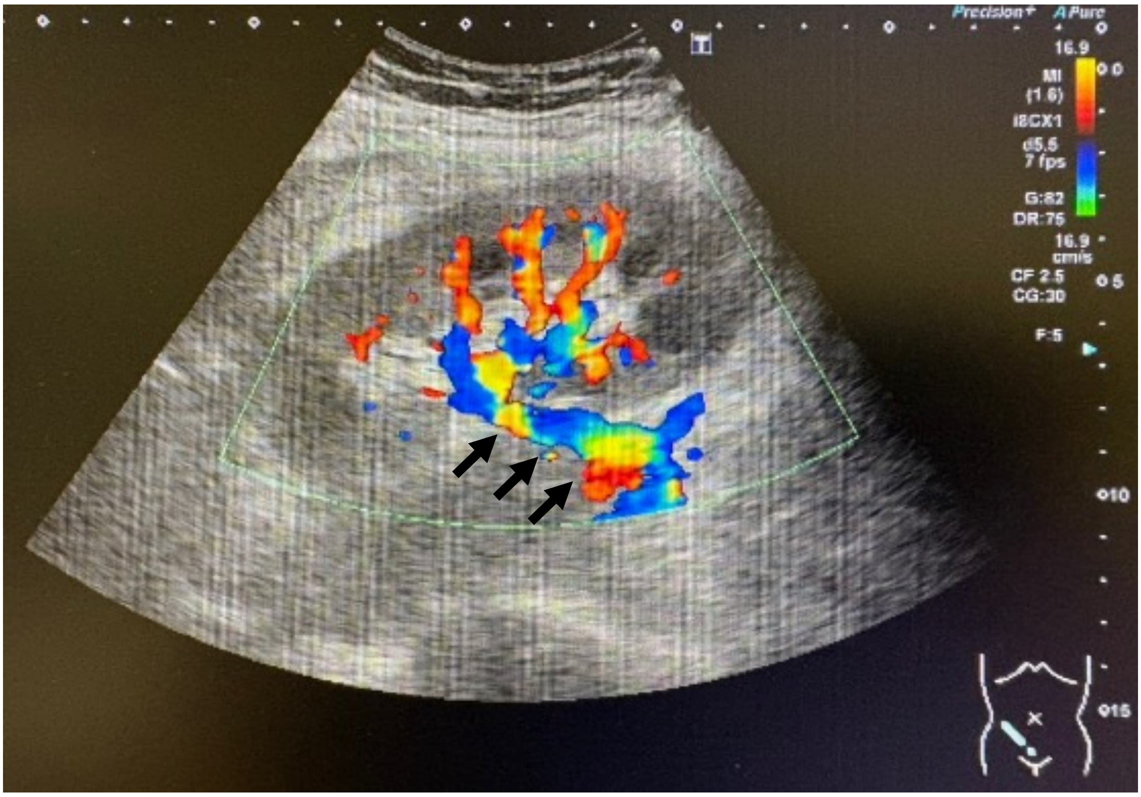

Supplemental Fig. S1: The ultrasound with color Doppler of the transplanted kidney on post operative day 21 shows no signs of renal artery stenosis (black arrows).
